# Targeting of cadherin-11 decreases skin fibrosis in the tight skin-1 mouse model

**DOI:** 10.1371/journal.pone.0187109

**Published:** 2017-11-07

**Authors:** Mesias Pedroza, Robert L. Welschhans, Sandeep K. Agarwal

**Affiliations:** Section of Immunology, Allergy, and Rheumatology, Biology of Inflammation Center, Department of Medicine, Baylor College of Medicine, Houston, TX, United States of America; University of Bergen, NORWAY

## Abstract

**Objective:**

Systemic sclerosis (SSc) is an autoimmune disease clinically manifesting as progressive fibrosis of the skin and internal organs. Cadherin-11 (CDH11) expression is increased in fibrotic skin and lung tissue. Targeting CDH11 may be an effective approach to treating fibrosis. We hypothesize that targeting CDH11 will decrease fibrosis in the tight skin-1 (Tsk-1) mouse model.

**Methods:**

CDH11 expression was determined in the Tsk-1 mouse model using quantitative real time PCR and immunofluorescence (IF). Inhibitory anti- CDH11 monoclonal antibodies were tested in Tsk-1 mice for their ability to decrease hypodermal fibrosis.

**Results:**

Expression of CDH11 was increased in fibrotic skin from Tsk-1 mice compared to pallid controls. IF staining demonstrated that CDH11 expression localized to fibroblasts within the hypodermis of fibrotic skin. Treatment with inhibitory anti-CDH11 monoclonal antibodies decreased hypodermal thickness and fibrotic mediators in Tsk-1 mice compared to control antibodies.

**Conclusions:**

These data demonstrate an important role for CDH11 in the development of skin fibrosis in Tsk-1 mice. These data add to the growing evidence for the important role of CDH11 in tissue fibrosis and fibrotic disease such as systemic sclerosis.

## Introduction

Scleroderma (systemic sclerosis, SSc) is an autoimmune disease clinically characterized by progressive fibrosis of the skin and internal organs. The mechanisms that lead to fibrosis in SSc involve three processes: vasculopathy, inflammation and autoimmunity, and excessive extracellular matrix (ECM) deposition. [1] At the cellular level, fibroblast and myofibroblasts are key producers of ECM.[1] At the molecular level, multiple pathways have been implicated in SSc including TGF-**β**, type I interferon, Wnt-**β** catenin and cadherins. [1–3]

Cadherins are transmembrane proteins that mediate calcium-dependent homophilic cell-to-cell adhesion.[4] The cytoplasmic tail of cadherins binds to β-catenin, linking the cadherin to the actin cytoskeleton through α. Cadherins play a role in regulating cellular behavior beyond adhesion. Specifically, cadherins are key regulator of cell migration and invasion.[5] Cadherins have also been implicated in regulating epithelial-to-mesenchymal transition (EMT) and myofibroblasts differentiation.[2, 6, 7]

Cadherin-11 (CDH11) is a type II classical cadherin.[8] CDH11 expression has been reported on mesenchymal cells including synovial, dermal and lung fibroblasts. [9–12] CDH11 contributes to the mesenchymal phenotype and regulates cellular invasion.[9–12] CDH11 expression is increased during the differentiation of fibroblasts to myofibroblasts and may reinforce cell-to-cell adhesion and contractility.[7, 13] A role for CDH11 has also been proposed in SSc and idiopathic pulmonary fibrosis (IPF). For example, expression of CDH11 is increased in SSc skin and IPF lungs, where it is observed on fibroblasts, myofibroblasts, and macrophages.[2, 14] Furthermore, CDH11 deficient mice develop less dermal and lung fibrosis induced by bleomycin.[2, 14] Finally, monoclonal antibodies targeting CDH11 decrease dermal and lung fibrosis induced by bleomycin. [2, 14]

Individual mouse models of dermal fibrosis do not adequately mimic all of the pathological features of SSc. Therefore, it is important to demonstrate a role for a candidate molecule in multiple models.[15, 16] For example, the bleomycin model is an inflammation-driven dermal fibrosis model resembling the early inflammatory stages of SSc.[17] In contrast tight skin-1 (Tsk-1) mice develop hypodermal fibrosis due to a tandem duplication of the fibrillin-1 (*Fbn-1*) gene. This results in an oversized Fbn-1 protein that leads to activation of TGF-**β** pathways, abnormal microfibril deposition, and thickening of the hypodermis. [18–20] In contrast to the bleomycin model, fibrosis in the Tsk-1 model is less dependent on inflammation. Both models complement each other and serve as tools to test anti-fibrotic agents in an inflammation-dependent and independent settings. Therefore, in the current manuscript we sought to extend our prior observations of a role for CDH11 in dermal fibrosis induced by bleomycin and determine the extent to which CDH11 contributes to the development of hypodermal fibrosis in the Tsk-1 mouse model.

## Methods

### Mice

Tsk-1 mice and pallid (pa/pa) controls were obtained from The Jackson Laboratory and colonies were maintained at BCM in microisolator caging systems in a specific pathogen free facility. For the current studies only female mice were studied. All studies were conducted with the approval of the BCM Institutional Animal Care and Use Committees (AN-6128). The current experiments were in accordance with the Animal Research: Reporting of *In Vivi* Experiments (ARRIVE) guidelines (S1 File).

### Anti-cadherin-11 monoclonal antibody treatment

Starting at 5 weeks of age, neutralizing anti-CDH11 monoclonal antibody (clone 13C2) or isotpype control were administered via intraperitoneal injection for 4 weeks similar to prior reports. [2, 12] Mice were injected with a loading dose of 500 μg of antibodies followed by 100 μg injections three times a week. At 9 weeks of age, skin biopsies from identical anatomic areas on the back, starting behind the front limbs and moving toward the tail, were obtained for analyses including histology, collagen content and tissue mRNA levels of fibrotic mediators.

### Histology

Five μm thick sections of paraffin-embedded skin were stained with hematoxylin and eosin or Masson’s Trichrome. Hypodermal thickness was used to quantify fibrosis by measuring the thickness of subcutaneous connective tissue beneath the panniculus carnosus at six randomly selected sites per microscopic field in each animal.

For immunofluorescence (IF), sections were incubated at 4°C overnight with Alexa Fluor 488-conjugated anti-CDH11 antibody (1:100 dilution, R&D systems), Alexa Fluor 647-conjugated anti-**α**SMA (1:200, dilution, Abcam), Alexa Fluor 647-conjugated anti-F40/80 (1:50 dilution, Abcam) or species-specific isotype antibodies (R&D systems and Abcam). Sections were mounted with ProLong Gold antifade reagent with DAPI (Life Technologies). Fibroblasts were identified in the hypodermis section by their spindle-shape morphology.

### Biochemical analysis of skin biopsies

The collagen content of the skin was determined by Sircol Collagen Assay kit (Biocolor, Newtown Abbey, UK).[21] Total protein assay (Bio-Rad Laboratories, Hercules, CA) was used as control to normalize collagen content of each sample.

Tissue mRNA levels were determined by real-time quantitative PCR (RT-PCR). Total RNA was isolated from skin frozen in RNA Later (Qiagen Sciences, Maryland, MA) and purified with RNA mini kit (Qiagen Sciences, Maryland, MA). Real-time PCR was performed using validated TaqMan Gene Expression Assays for *Cdh11*, *Col1***α***1*, alpha-smooth muscle actin (**α***SMA)*, *CCN2 (formerly called connective tissue growth factor)*, *fibronectin*, *TGF-***β***1*, *IL-6*, and *18s* RNA (Applied Biosystems). The *18s* RNA gene was used as a control to normalize transcript levels of mRNA in each sample. Data are presented as fold change.

### Statistical analysis

Results are expressed as the means ± SD or ± SEM. Mann-Whitney’s U-test was used for comparison between two groups. P values < 0.05 were considered statistically significant.

## Results

### Cadherin-11 expression in TSK-1 mice

**Fig 1:**
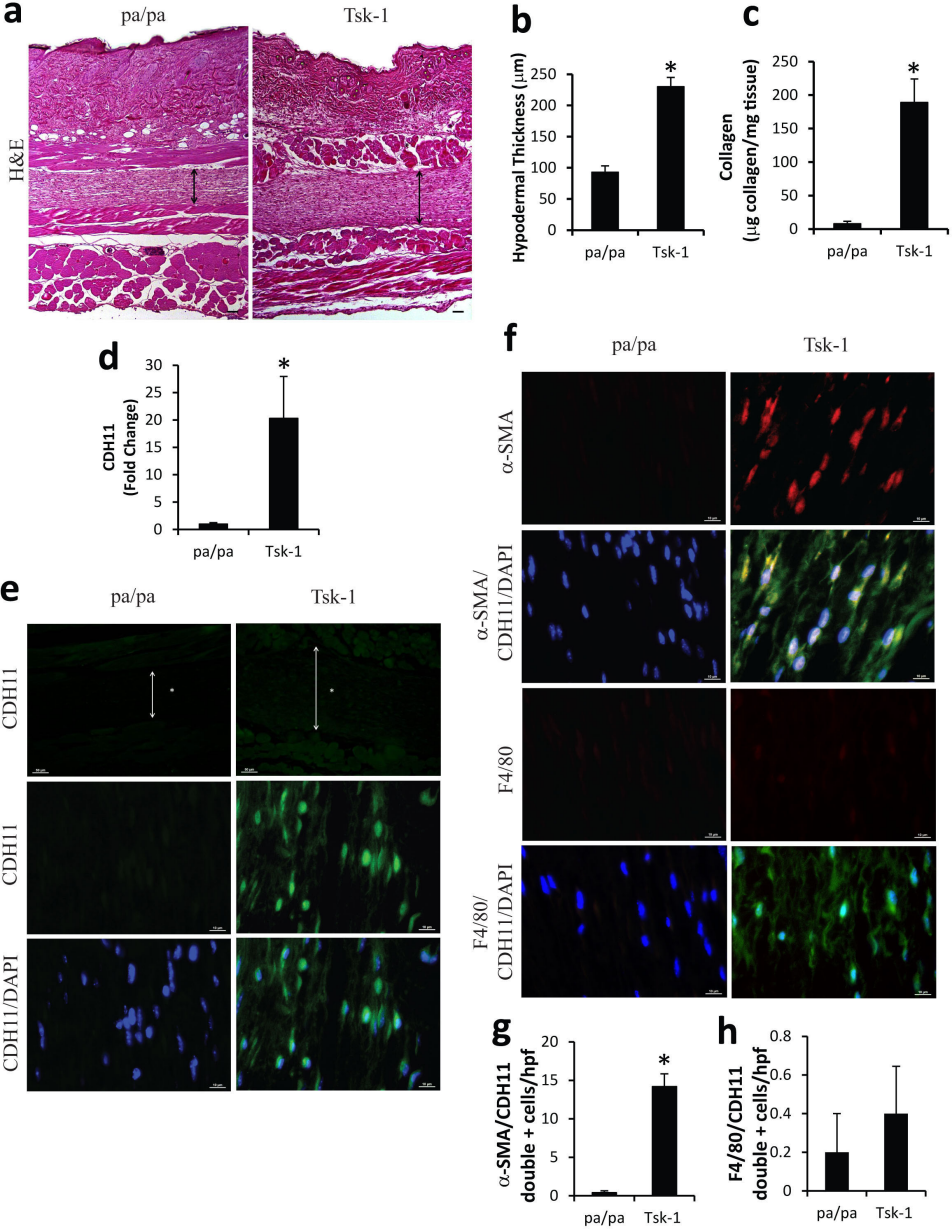
Characterization of Cadherin-11 expression in the Tsk-1 mouse model of skin fibrosis. (a) Skin biopsies from pa/pa and Tsk-1 mice were stained with H&E demonstrating increased thickness of the hypodermis (arrow) in Tsk-1 mice compared to pa/pa mice. (10x magnification, scale bar 50 μm). These data were quantified in (b) (n = 10; * p ≤ 0.05 pa/pa vs. Tsk-1). (c) Tsk-1 mice have increased deposition of collagen using Sircol assay (n = 10; * p ≤ 0.05 pa/pa vs. Tsk-1). (d) Skin biopsies were used to isolate total RNA and transcripts were determined for *Cdh11*. Transcripts were measured in parallel with *18S* RNA and values are presented as mean of fold change transcripts ± SEM, n = 10 (* p ≤ 0.05 pa/pa vs. Tsk-1). (e) Immunofluorescence of CDH11 expression was determined in the hypodermal layer (arrows) in skin sections from pa/pa and Tsk-1 mice. Images are representative of 10 mice from each group. Top panels, Low magnification 20X, scale bars: 50 μm. Middle and lower panel, high magnification 100x, scale bars: 10 μm. (f) Dual color IF of hypodermis from pa/pa and Tsk-1 mice stained for **α**SMA (red) and CDH11 (green) or F4/80 (red) and CDH11 (green) demonstrate that CDH11 expression primarily localizes to **α**SMA myofibroblasts (100x, scale bars: 10 μm). (g, h) Quantification of **α**SMA and F4/80 positive cells that express CDH11 per high power field in the hypodermis of pa/pa and Tsk-1 mice (* p ≤ 0.05 pa/pa vs. Tsk-1).

To determine if CDH11 expression is increased in fibrotic skin in Tsk-1 mice, total RNA was isolated from skin biopsies of Tsk-1 mice and pa/pa control mice. As seen in Fig 1, Tsk-1 mice have an increase in the thickness of the hypodermis (Fig 1A and 1B) and increase in collagen content as assessed by Sircol assay (Fig 1C), relative to the pa/pa control. Using relative qRTPCR it was observed that *Cdh11* mRNA levels are increased in Tsk-1 mice compared to pa/pa controls (Fig 1D). Skin biopsies were also processed for IF analyses to determine the cellular localization of CDH11 expression. As seen in Fig 1E, expression of CDH11 was low in pa/pa mice. In contrast, CDH11 was abundantly expressed on cells primarily located in the hypodermis of Tsk-1 mice. To better determine the cellular source of CHD11 production, dual color IF was performed (Fig 1F). Tsk-1 mice had an increase in the number of myofibroblasts that expressed **α**SMA. These cells also stained positive for CDH11. In contrast, very few macrophages were identified in the hypodermis of both pa/pa control mice and Tsk-1 mice. These data were quantified by counting the number of **α**SMA and F4/80 positive cells that stained for CDH11 in pa/pa and Tsk-1 skin biopsies (Fig 1G and 1H). Together these data demonstrate that CDH11 levels are increased in Tsk-1 skin and localize to myofibroblasts in the hypodermal layer.

### Reduction in skin fibrosis with anti-cadherin-11 antibody treatment

**Fig 2:**
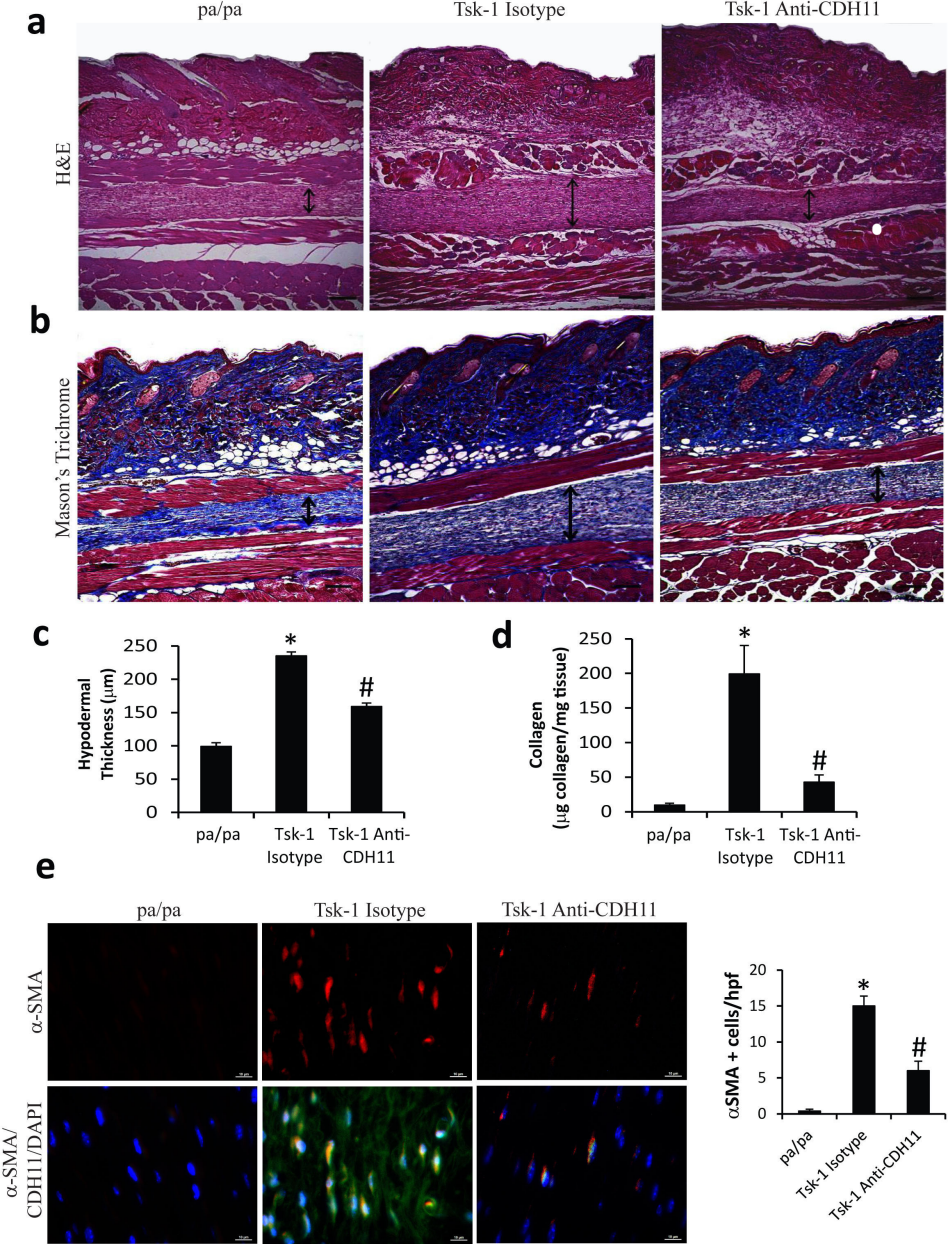
Decreased hypodermal fibrosis in Tsk-1 mice after Cadherin-11 antibody treatment. Examination of skin histology through H&E staining (a) and Masson’s Trichrome (b) from pa/pa control mice (left), Tsk-1 mice treated with isotype antibodies (middle), and Tsk-1 mice treated with antibodies against CDH11 (13C2; right). These images demonstrated that neutralization of CDH11 reduces hypodermal fibrosis. Images are representative of 10 mice from each group. (c) Hypodermal thickness was measured to determine the amount of hypodermal fibrosis. (d) Soluble collagen protein levels were measured using Sircol Assay from skin samples obtained from pa/pa control mice and Tsk-1 mice treated with isotype or anti-CDH11 antibodies. (e) Dual color IF (CDH11 in green and **α**SMA in red) of hypodermis from pa/pa and Tsk-1 mice treated with isotype of anti-CDH11 antibodies. Anti-CDH11 antibodies decreased **α**SMA positive cells in Tsk-1 mice. Data are presented as mean ± SEM, n = 10 (*p ≤ 0.05 pa/pa vs. Tsk-1 isotype; #p ≤ 0.05 Tsk-1 isotype vs. Tsk-1 anti-CDH11).

CDH11 plays a role in the development of dermal fibrosis induced by bleomycin.[14] Since CDH11 expression is increased in the Tsk-1 skin, we hypothesized that CDH11 is a mediator of skin fibrosis in the Tsk-1 model. Anti-CDH11 monoclonal antibody (clone 13C2) was administered to Tsk-1 and pa/pa mice starting at 5 weeks of age. 13C2 has been reported to inhibit CDH11 in mouse models of inflammatory arthritis and lung fibrosis.[2, 12] Mice tolerated injections with either anti-CDH11 antibody or isotype antibodies without any overt signs of toxicity, appearance or behavior. Representative histological images stained with H&E (Fig 2A) and Masson’s Trichrome (Fig 2B) demonstrate that anti-CDH11 monoclonal antibody treated Tsk-1 mice have less hypodermal fibrosis compared to isotype treated mice. Quantitative assessment of hypodermal thickness (Fig 2C) demonstrate that Tsk-1 mice have increased hypodermal thickness relative to pa/pa control mice which was unaffected by isotype control antibodies. Interestingly, anti-CDH11 monoclonal antibody treatment decreased hypodermal thickness in Tsk-1 mice. These data were further confirmed using Sircol assay to assess total collagen deposition in lesional skin (Fig 2D). Collagen accumulation in Tsk-1 mice was significantly reduced with treatment of anti-CDH11 monoclonal antibody relative to isotype. Finally dual color IF for **α**SMA and CDH11 was used to identify myofibroblasts in the hypodermis (Fig 2E). Consistent with a decrease in fibrosis, Tsk-1 mice treated with anti-CDH11 monoclonal antibodies had fewer cells stain positive for **α**SMA compared to Tsk-1 mice treated with isotype control antibodies. Together these data demonstrate that inhibition of CDH11 with a monoclonal antibody decreased hypodermal fibrosis in Tsk-1 mice.

**Fig 3:**
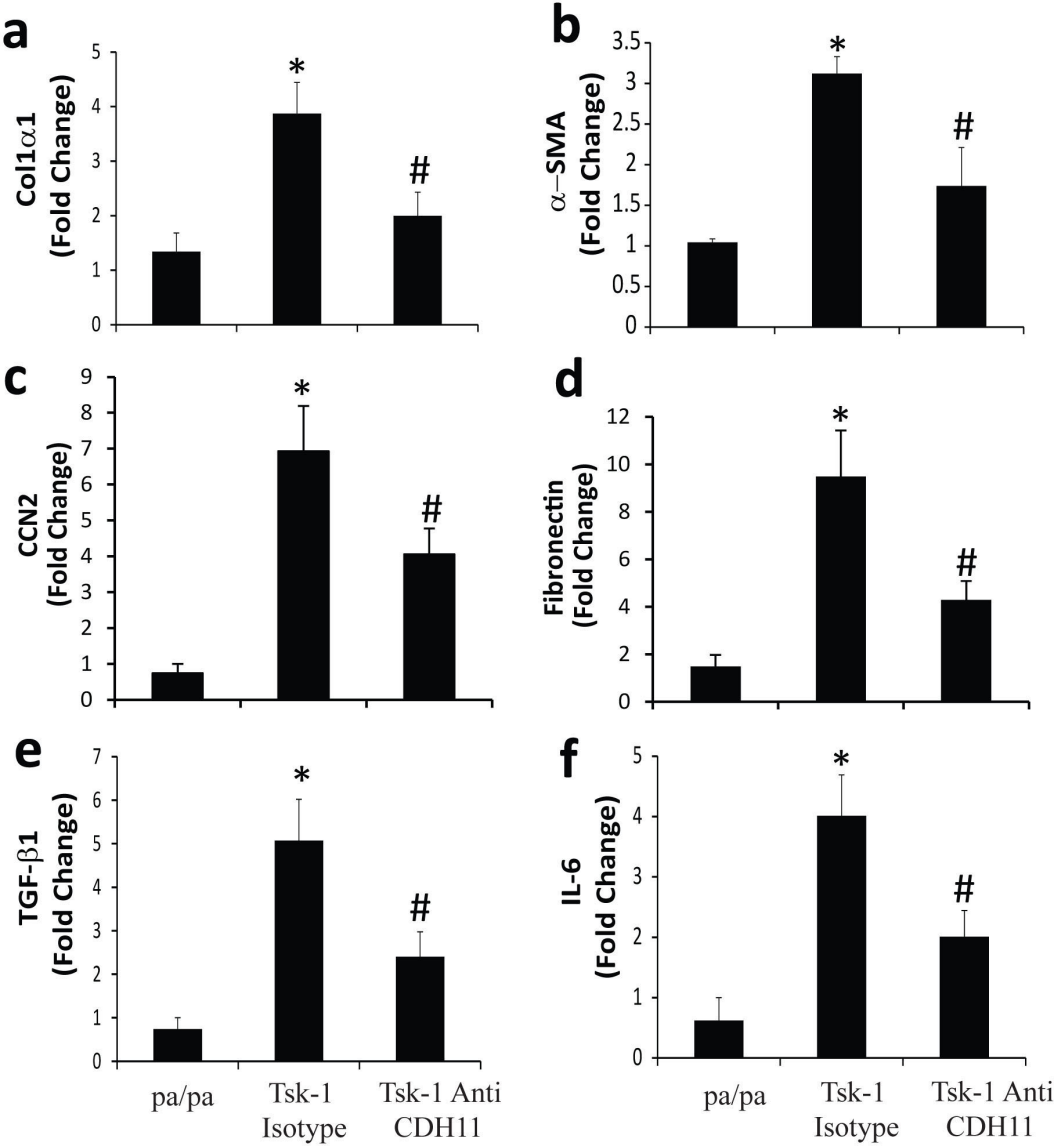
Anti-cadherin-11 antibody decreases mediators of fibrosis in Tsk-1 mice. Total RNA was isolated from skin biopsies from pa/pa control mice, Tsk-1 mice treated with isotype antibodies, and Tsk-1 mice treated with antibodies against CDH11 (13C2). Transcripts were determined for *Col1***α***1* (a), **α***SMA* (b), *CCN2* (c), fibronectin (d), *TGF-***β** (e), *IL-6* (f) in parallel with *18S* rRNA. Data are presented as mean of fold change transcripts ± SEM, n≥10 (*p ≤ 0.05 pa/pa vs. Tsk-1 isotype; #p ≤ 0.05 Tsk-1 isotype vs. Tsk-1 anti-CDH11).

Total RNA was isolated from skin biopsies of Tsk-1 mice treated with isotype control or anti-CDH11 antibodies to further quantify fibrotic endpoints in the Tsk-1 mice using quantitative real time PCR. As seen in Fig 3A–3F, skin from Tsk-1 mice treated with isotype control had increased expression of *Col1***α***1*, **α***SMA*, *CCN2*, fibronectin and TGF-**β** compared to pa/pa control mice. Treatment with anti-CDH11 antibodies decreased the expression of *Col1***α***1*, **α***SMA*, *CCN2*, fibronectin and TGF-**β** relative to isotype treated mice. Interestingly, IL-6, a pleiotropic pro-fibrotic cytokine, is also increased in Tsk-1 skin and subsequently reduced by anti-CDH11 antibody treatment. Together these data demonstrate that CDH11 plays an important role in development of skin fibrosis in the Tsk-1 mouse model.

## Discussion

In this concise report, two important observations were made. First, CDH11 expression is increased in fibrotic skin from Tsk-1 mice, localizing to hypodermal myofibroblasts. Second, inhibition of CDH11 using anti-CDH11 monoclonal antibodies is effective in reducing quantitative measures of hypodermal fibrosis in Tsk-1 mice.

The development of tissue fibrosis in patients with SSc is a complex process involving endothelial cell dysfunction and inflammatory cells (macrophage) activation that lead to the upregulation of pro-fibrotic cytokines such as TGF-**β**.[1] Dermal (or lung) fibroblasts and myofibroblasts accumulate in the tissue, leading to increased ECM deposition and fibrosis. Understanding the molecular pathways involved in the development of fibrosis using multiple animal models is critical to the development of therapeutic targets for fibrotic diseases such as SSc.

CDH11 is increased in affected skin of SSc patients and the levels correlate with the extent of skin involvement and the expression of TGF-**β** related genes.[14] Furthermore, CDH11 regulates the development of skin and lung fibrosis induced by bleomycin.[2, 14] The bleomycin models are dependent on tissue injury and inflammation. Based on the expression pattern of CDH11 in these models, it is possible that CDH11 regulates the fibrotic phase but also tissue inflammation.[12] In contrast, the Tsk-1 model is less dependent on inflammation and more directly dependent on TGF-**β** and fibroblast activation. Therefore this presents the opportunity to investigate if CDH11 more directly regulates the fibroblast response in vivo. Indeed, in the current report, the ability of anti-CDH11 monoclonal antibodies to decrease the hypodermal fibrosis seen in the Tsk-1 model indicates that CDH11 can regulate the development of tissue fibrosis through the TGF-**β** pathway and the fibroblast, independent of its role in the regulation of inflammation. However these do not rule out the possibility that CDH11 also regulates inflammation in the context of other disease models.

The importance of CDH11 in fibroblast behavior has been demonstrated in synovial fibroblasts where it is important for migration and may also induce IL-6.[12, 22] CDH11 also has been shown to regulate dermal fibroblast migration and invasion.[14] CDH11 expression is increased during the differentiation of fibroblasts to myofibroblasts and may reinforce intercellular contacts and contractility.[7, 13] Furthermore, CDH11 has recently been shown to regulate tissue mechanics and extracellular matrix production.[23] These observations are consistent with our current in vivo data, suggesting CDH11 regulates the dermal fibroblast *in vivo*. On going studies are focusing on the relative contribution of CDH11 to the macrophage and fibroblast in the context of fibrosis.

The current manuscript adds to the growing evidence that points towards the importance of CDH11 in tissue fibrosis and has important clinical implications SSc and fibrotic diseases. The ability of CDH11 inhibition to decrease and/or treat fibrosis in multiple murine models of fibrosis suggests CDH11 is a potential therapeutic target in SSc. Future studies on CDH11 in SSc are needed to advance our knowledge of CDH11 in fibrosis and its clinical translation.

## Supporting information

S1 File Research: Reporting of *In Vivi* experiments (ARRIVE) checklist. (PDF)
